# Prognostic model for predicting outcome and guiding treatment decision for unresectable hepatocellular carcinoma treated with lenvatinib monotherapy or lenvatinib plus immunotherapy

**DOI:** 10.3389/fimmu.2023.1141199

**Published:** 2023-02-23

**Authors:** De-Zhen Guo, Shi-Yu Zhang, San-Yuan Dong, Jia-Yan Yan, Yu-Peng Wang, Ya Cao, Sheng-Xiang Rao, Jia Fan, Xin-Rong Yang, Ao Huang, Jian Zhou

**Affiliations:** ^1^ Department of Liver Surgery and Transplantation, Liver Cancer Institute, Zhongshan Hospital, Fudan University, Shanghai, China; ^2^ Key Laboratory of Carcinogenesis and Cancer Invasion, Ministry of Education, Fudan University, Shanghai, China; ^3^ Shanghai Key Laboratory of Organ Transplantation, Zhongshan Hospital, Fudan University, Shanghai, China; ^4^ Department of Radiology, Zhongshan Hospital, Fudan University Shanghai Institute of Medical Imaging, Shanghai, China; ^5^ Cancer Research Institute, Central South University, Changsha, China; ^6^ Key Laboratory of Carcinogenesis and Cancer Invasion, Ministry of Education, Changsha, China; ^7^ Institute of Biomedical Sciences, Fudan University, Shanghai, China; ^8^ State Key Laboratory of Genetic Engineering, Fudan University, Shanghai, China

**Keywords:** predicting model, liver cancer, lenvatinib, immunotherapy, protein induced by vitamin K absence or antagonist-II

## Abstract

**Background:**

Lenvatinib monotherapy and combination therapy with immune checkpoint inhibitors (ICI) were widely applied for unresectable hepatocellular carcinoma (uHCC). However, many patients failed to benefit from the treatments. A prognostic model was needed to predict the treatment outcomes and guide clinical decisions.

**Methods:**

304 patients receiving lenvatinib monotherapy or lenvatinib plus ICI for uHCC were retrospectively included. The risk factors derived from the multivariate analysis were used to construct the predictive model. The C-index and area under the receiver-operating characteristic curve (AUC) were calculated to assess the predictive efficiency.

**Results:**

Multivariate analysis revealed that protein induced by vitamin K absence or antagonist-II (PIVKA-II) (HR, 2.05; P=0.001) and metastasis (HR, 2.07; P<0.001) were independent risk factors of overall survival (OS) in the training cohort. Herein, we constructed a prognostic model called PIMET score and stratified patients into the PIMET-low group (without metastasis and PIVKA-II<600 mAU/mL), PIMET-int group (with metastasis or PIVKA-II>600 mAU/mL) and PIMET-high group (with metastasis and PIVKA-II>600 mAU/mL). The C-index of PIMET score for the survival prediction was 0.63 and 0.67 in the training and validation cohort, respectively. In the training cohort, the AUC of 12-, 18-, and 24-month OS was 0.661, 0.682, and 0.744, respectively. The prognostic performances of the model were subsequently validated. The AUC of 12-, 18-, and 24-month OS was 0.724, 0.726, and 0.762 in the validation cohort. Subgroup analyses showed consistent predictive value for patients receiving lenvatinib monotherapy and patients receiving lenvatinib plus ICI. The PIMET score could also distinguish patients with different treatment responses. Notably, the combination of lenvatinib and ICI conferred survival benefits to patients with PIMET-int or PIMET-high, instead of patients with PIMET-low.

**Conclusion:**

The PIMET score comprising metastasis and PIVKA-II could serve as a helpful prognostic model for uHCC receiving lenvatinib monotherapy or lenvatinib plus ICI. The PIMET score could guide the treatment decision and facilitate precision medicine for uHCC patients.

## Introduction

Hepatocellular carcinoma (HCC) is a common malignancy worldwide and a leading cause of cancer-related death (1, 2). Patients with HCC are frequently diagnosed at an advanced stage and are not eligible for curative treatments such as surgical resection, liver transplantation, or ablation, leading to a poor prognosis (3, 4).

Recently, lenvatinib, a tyrosine kinase inhibitor, showed favorable efficacy for advanced HCC and was approved as one of the first-line therapy by the FDA (5). Furthermore, the combination of lenvatinib and immune checkpoint inhibitors (ICI) showed improved efficacy in several malignancies (6–9). However, many patients with unresectable HCC (uHCC) failed to benefit from these treatments. Several prognostic models were established to predict the efficacies and outcomes of treatments (10–13). However, majority of models were built only for patients receiving lenvatinib monotherapy or immunotherapy, which limited their application. A prognostic model to predict the outcomes of both lenvatinib monotherapy and combination therapy of lenvatinib and ICI was lacking.

In addition, the combination of lenvatinib and ICI failed to show the superior efficacy in HCC compared with lenvatinib monotherapy in the phase III clinical trial LEAP-002, indicating the necessity of patient selection for the combination therapy. A model with the ability to identify patients with uHCC who might benefit from lenvatinib plus ICI could help patient selection and guide treatment decisions.

In this study, we constructed a prognostic model for patients with uHCC receiving lenvatinib monotherapy or lenvatinib plus ICI. Importantly, the newly developed model could identify the patients who might benefit from lenvatinib plus ICI and guide the clinical treatment decisions.

## Materials and methods

### Patients

A total of 304 patients treated with lenvatinib monotherapy or lenvatinib plus ICI as first-line treatment for uHCC at Zhongshan Hospital between October 2018 and December 2020 were retrospectively included. The inclusion criteria were: clinically diagnosed as HCC; the Barcelona Clinic Liver Cancer (BCLC) B-C stage; lenvatinib for more than one month; at least one imaginary follow-up; complete baseline information. Tumor differentiation was assessed using the Edmondson grading system and liver function was evaluated using the Child-Pugh scoring system. The BCLC system (14) and The Guidelines of Primary Liver Cancer in China (15) were used to determine the tumor stage. To avoid potential bias, the included patients were randomly assigned to the training cohort and validation cohort at a 2:1 ratio. The research was conducted in accordance with the declaration of Helsinki and ethical approvals were obtained from the ethics committee of Zhongshan hospital (B2020-401).

### Treatments and assessments

Lenvatinib (Levima^®^, Eisai, Tokyo, Japan) was administered orally to most patients at a dose of either 8 mg/day for patients <60 kg or 12 mg/day for patients >60kg. The withdrawal was determined when disease progression, unbearable adverse events, or personal reasons. For the patients receiving lenvatinib plus ICI, immunotherapy began on the first day of lenvatinib and was performed every three weeks afterward. The decision to treat patients with lenvatinib monotherapy or lenvatinib plus ICI was made by the multidisciplinary tumor board. The combination therapy of lenvatinib plus ICI was recommended to patients with uHCC. The patients with contraindication of ICI or refused ICI due to high cost or other personal reasons were treated with lenvatinib monotherapy.

Patients were follow-up *via* enhanced computed tomography or magnetic resonance imaging in the first month and then every three months after the initiation of treatment. The best tumor response was assessed in the standard of modified Response Evaluation Criteria in Solid Tumors (16). The objective response rate (ORR) was defined as the rate of patients with complete response or partial response and the disease control rate (DCR) was defined as the proportion of complete response, partial response or stable disease. Progression-free survival (PFS) was defined as the interval from initiation of treatment to progression or death from any cause. Overall survival (OS) was defined as the interval from initiation of treatment to death from any cause. The date of the last follow-up was December 1st, 2022.

### Statistical analyses

The baseline was compared using the chi-square and Fisher exact test. Continuous variables were summarized as median (interquartile range) and the Wilcoxon rank sum test was applied for comparison. Kaplan-Meier was performed to calculate OS and PFS in different groups and the log-rank test was applied to compute the significance. Cox regression analysis was performed to identify the risk factor of patients with HCC, and the variables with P <0.05 were applied to develop the prognostic model in the training cohort. To evaluate the efficiency of the model, the C-statistics and the time-dependent area under the receiver-operating characteristic curve (AUC) values were calculated. All statistical tests were two-tailed and a P value <0.05 was considered statistically significant. The statistics were performed using R software 4.1.2 (R Foundation for Statistical Computing, Vienna, Austria).

## Results

### Patient characteristics

**Figure 1:**
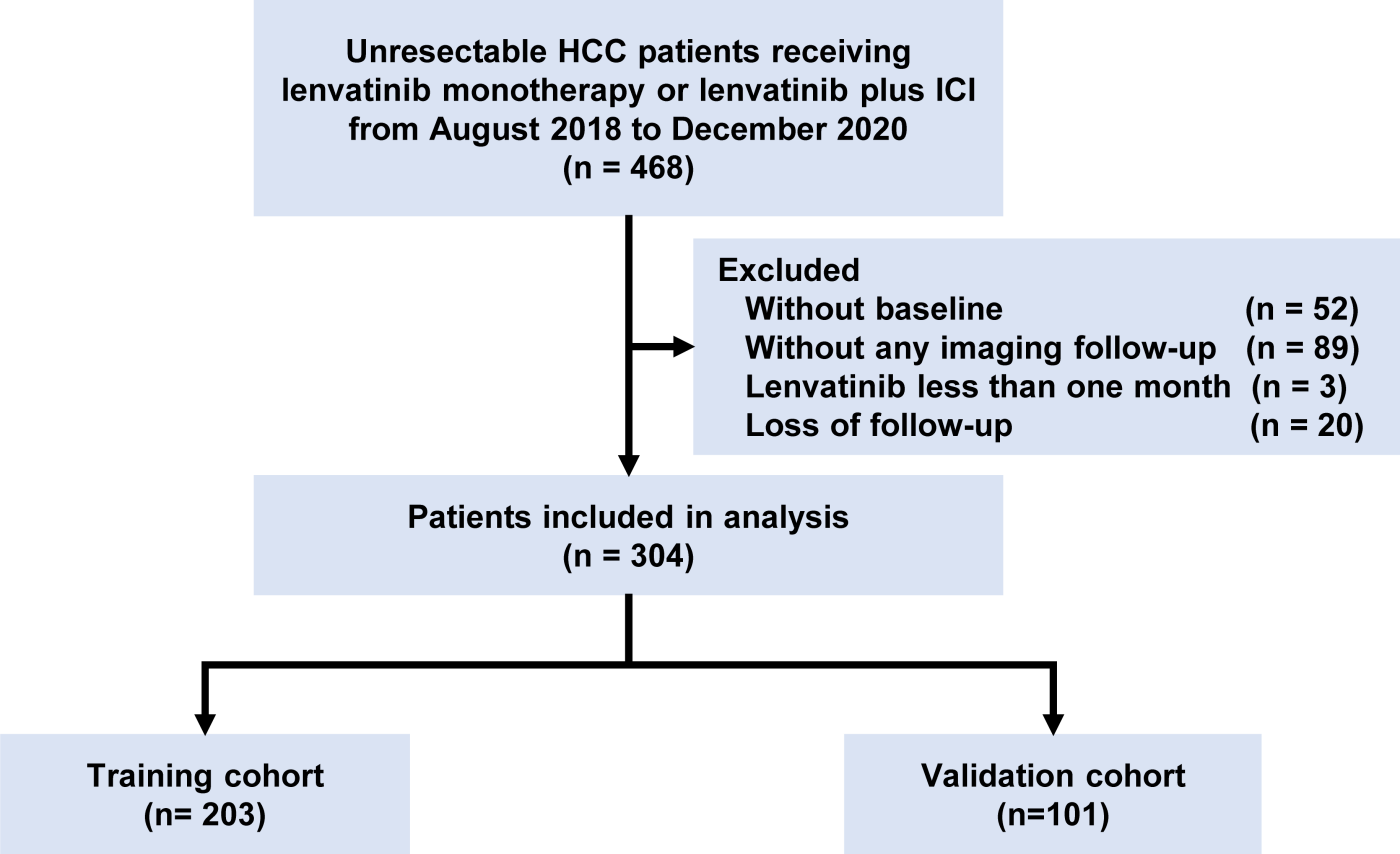
Flowchart of the study. HCC, hepatocellular carcinoma.

A total of 304 patients receiving lenvatinib monotherapy or combination therapy with ICI were analyzed, including 203 and 101 in the training cohort and validation cohort, respectively (**Figure 1**). Baseline characteristics were shown in **Table 1** and there was no significant difference between the training and validation cohort. Most of the patients were Child-Pugh A stage (280, 92.1%) and BCLC C stage (247, 81.2%). There were 183 (60.2%) patients with macrovascular invasion (MVI) and 122 (40.1%) with metastasis. There were 113 (37.2%) patients treated with lenvatinib monotherapy and 191 (62.8%) patients treated with lenvatinib plus ICI, including camrelizumab (60, 31.4%), nivolumab (8, 4.2%), pembrolizumab (29, 15.2%), sintilimab (52, 27.2%), tislelizumab (6, 3.1%), and toripalimab (36, 18.8%). The median treatment cycle of ICI was 9 (range, 1-31). The median duration of follow-up was 15.5 (interquartile range, 8.8-22.7) months. 229 (75.3%) patients died during the follow-up.

**Table 1:** Baseline characteristics.

	Level	The whole cohort (n=304)	Training cohort (n=203)	Validation cohort (n=101)	P
**Gender**	Male	268 (88.2)	180 (88.7)	88 (87.1)	0.839
	Female	36 (11.8)	23 (11.3)	13 (12.9)	
**Age (years)**	≤50	100 (32.9)	65 (32.0)	35 (34.7)	0.741
	>50	204 (67.1)	138 (68.0)	66 (65.3)	
**Etiology**	HBV	261 (85.9)	174 (85.7)	87 (86.1)	1
	Other	43 (14.1)	29 (14.3)	14 (13.9)	
**ECOG**	0 score	246 (80.9)	165 (81.3)	81 (80.2)	0.943
	1 score	58 (19.1)	38 (18.7)	20 (19.8)	
**Child-Pugh stage**	A	280 (92.1)	187 (92.1)	93 (92.1)	1
	B	24 (7.9)	16 (7.9)	8 (7.9)	
**Tumor size (cm)**	≤ 10cm	216 (71.1)	140 (69.0)	76 (75.2)	0.316
	> 10cm	88 (28.9)	63 (31.0)	25 (24.8)	
**Tumor number**	1 lesion	102 (33.6)	64 (31.5)	38 (37.6)	0.449
	2-3 lesions	48 (15.8)	35 (17.2)	13 (12.9)	
	>3 lesions	154 (50.7)	104 (51.2)	50 (49.5)	
**BCLC stage**	B	57 (18.8)	34 (16.7)	23 (22.8)	0.266
	C	247 (81.2)	169 (83.3)	78 (77.2)	
**MVI**	No	121 (39.8)	74 (36.5)	47 (46.5)	0.117
	Yes	183 (60.2)	129 (63.5)	54 (53.5)	
**Metastasis**	No	182 (59.9)	123 (60.6)	59 (58.4)	0.810
	Yes	122 (40.1)	80 (39.4)	42 (41.6)	
**AFP (ng/mL)**	(median[IQR])	366.00 [11.25,8789.25]	285.00 [10.95,7966.00]	466.00 [14.60,9424.00]	0.515
**PIVKA-II (mAU/mL)**	(median[IQR])	3332.50 [209.75,18917.75]	3873.00 [276.50,21632.00]	2721.00 [192.00,16599.00]	0.472

Data are reported as n (%).

AFP, alpha-fetoprotein; BCLC stage, Barcelona Clinic Liver Cancer; ECOG, Eastern Cooperative Oncology Group; HBV, hepatitis B virus; MVI, macrovascular invasion; PIVKA-II, Protein Induced by Vitamin K Absence or Antagonist-II.

### Construction of the PIMET score

**Figure 2:**
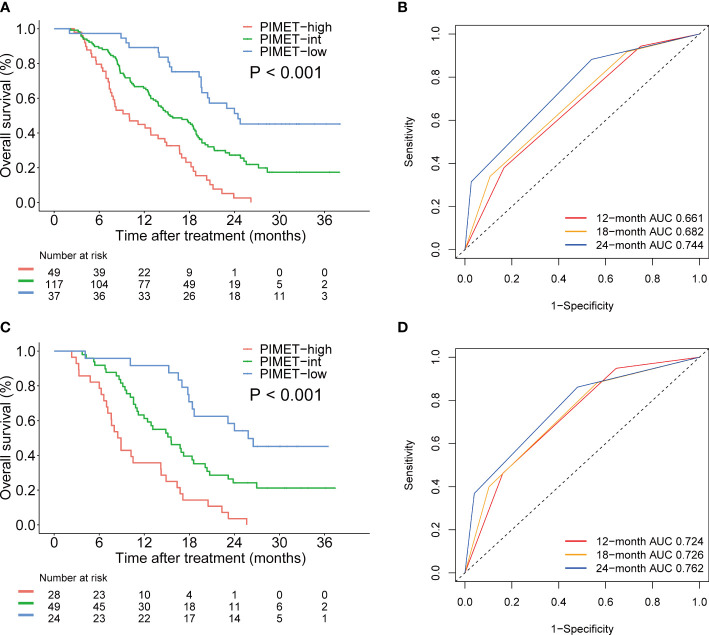
The prognostic efficiency of PIMET score for patients receiving lenvatinib monotherapy or lenvatinib plus ICI for unresectable HCC. **(A)** The Kaplan-Meier survival curves of OS according to PIMET score in the training cohort. **(B)** The AUCs of PIMET score for predicting 12-, 18-, and 24-month OS in the training cohort. **(C)** The Kaplan-Meier survival curves of OS according to PIMET score in the validation cohort. **(D)** The AUCs of PIMET score for predicting 12-, 18-, and 24-month OS in the validation cohort. AUC, area under the receiver-operating characteristic curve; HCC, hepatocellular carcinoma; OS, overall survival.

To develop a prognostic model for uHCC patients, we first performed univariate and multivariate Cox regression analyses in the training cohort (**Table 2**). It was identified that vitamin K absence or antagonist-II (PIVKA-II) >600 mAU/mL was an independent risk factor for uHCC patients (HR, 2.05, CI, 1.36-3.08, P=0.001). In addition, metastasis was another independent risk factor (HR, 2.07, CI, 1.49-2.87, P<0.001). Afterward, we constructed a prognostic model using PIVKA-II and metastasis. Since the coefficient values were similar between PIVKA-II (0.72) and metastasis (0.73), we developed an easy-to-use model based on those two variables and assigned 1 point for PIVKA-II>600 mAU/mL and 1 point for the presence of metastasis. The model was named the PIMET (PIVKA-II and METastasis) score. Patients were assigned into the PIMET-low group (0 points, without metastasis and PIVKA-II<600 mAU/mL), PIMET-int group (1 point, with metastasis or PIVKA-II>600 mAU/mL), or PIMET-high group (2 points, with metastasis and PIVKA-II>600 mAU/mL), respectively. In the training cohort, the C-index of the PIMET score was 0.63 and 0.56 for predicting the survival and progression of patients with uHCC. The median OS of patients in the PIMET-low group, PIMET-int group, and PIMET-high group was 24.5, 15.4, and 10.0 months, respectively. There was a significantly different OS among the three groups (P<0.001; **Figure 2A**). The AUC for 12-, 18-, and 24-month OS was 0.661, 0.682, and 0.744, respectively (**Figure 2B**). The PFS of patients in the PIMET-low group, PIMET-int group, and PIMET-high group also significantly differed (median PFS: 9.30 vs 6.67 vs 4.83 months; P=0.011; **Supplementary Figure 1A**). The AUC for 6-,12-, and 24-month PFS was 0.580, 0.626, and 0.730, respectively (**Supplementary Figure 1B**).

**Table 2:** Univariate and multivariate competing risk analyses to identify independent risk factors of OS in patients with unresectable HCC treated with lenvatinib monotherapy or lenvatinib plus ICI.

Variable	Univariate analysis		Multivariate analysis	
	HR (95% CI)	*P*	HR (95% CI)	*P*
**Gender (female)**	0.84 (0.51-1.39)	0.497	NA	NA
**Age (>50 years)**	0.86 (0.62-1.21)	0.389	NA	NA
**Etiology (HBV)**	0.55 (0.33-0.93)	0.025	0.74 (0.43-1.27)	0.277
**ECOG (1 score)**	0.75 (0.49-1.15)	0.188	NA	NA
**Child-Pugh stage (B)**	1.27 (0.72-2.25)	0.408	NA	NA
**Tumor size (> 10 cm)**	1.47 (1.05-2.07)	0.026	1.11 (0.77-1.61)	0.569
**Tumor number** **(>3 lesions)**	0.93 (0.78-1.11)	0.430	NA	NA
**MVI**	1.27 (0.91-1.78)	0.156	NA	NA
**Metastasis**	1.82 (1.32-2.51)	<0.001	2.07 (1.49-2.87)	<0.001
**AFP (> 100ng/mL)**	1.47 (1.06-2.04)	0.020	1.22 (0.87-1.71)	0.254
**PIVKA-II** **(>600 mAU/mL)**	2.17 (1.51-3.13)	<0.001	2.05 (1.36-3.08)	0.001

AFP, alpha-fetoprotein; BCLC stage, Barcelona Clinic Liver Cancer; CI, confidence interval; ECOG, Eastern Cooperative Oncology Group; HBV, hepatitis B virus; HCC, hepatocellular carcinoma; HR, hazard ratio; NA, not applicable; MVI, macrovascular invasion; PIVKA-II, Protein Induced by Vitamin K Absence or Antagonist-II.

### Validation of the PIMET score

Furthermore, the predictive efficiency of the model was verified and patients in the validation cohort were divided into the PIMET-low group, PIMET-int group, and PIMET-high group accordingly. The C-index for OS and PFS in the validation cohort was 0.67 and 0.61, respectively. The OS of patients with PIMET-low, PIMET-int, and PIMET-high were remarkably different (median OS: 25.8 vs 15.6 vs 8.7 months, P<0.001, **Figure 2C**) in the validation cohort. The AUC for 12-, 18-, and 24-month OS was 0.724, 0.726, and 0.762, respectively (**Figure 2D**). There were significantly different PFS among patients with different groups (median PFS: 10.6 vs 7.2 vs 4.9 months, P<0.001, **Supplementary Figure 1C**). The AUC for 6-, 12-, and 24-month PFS was 0.691, 0.674, and 0.781, respectively (**Supplementary Figure 1D**).

### Subgroup analysis

**Figure 3:**
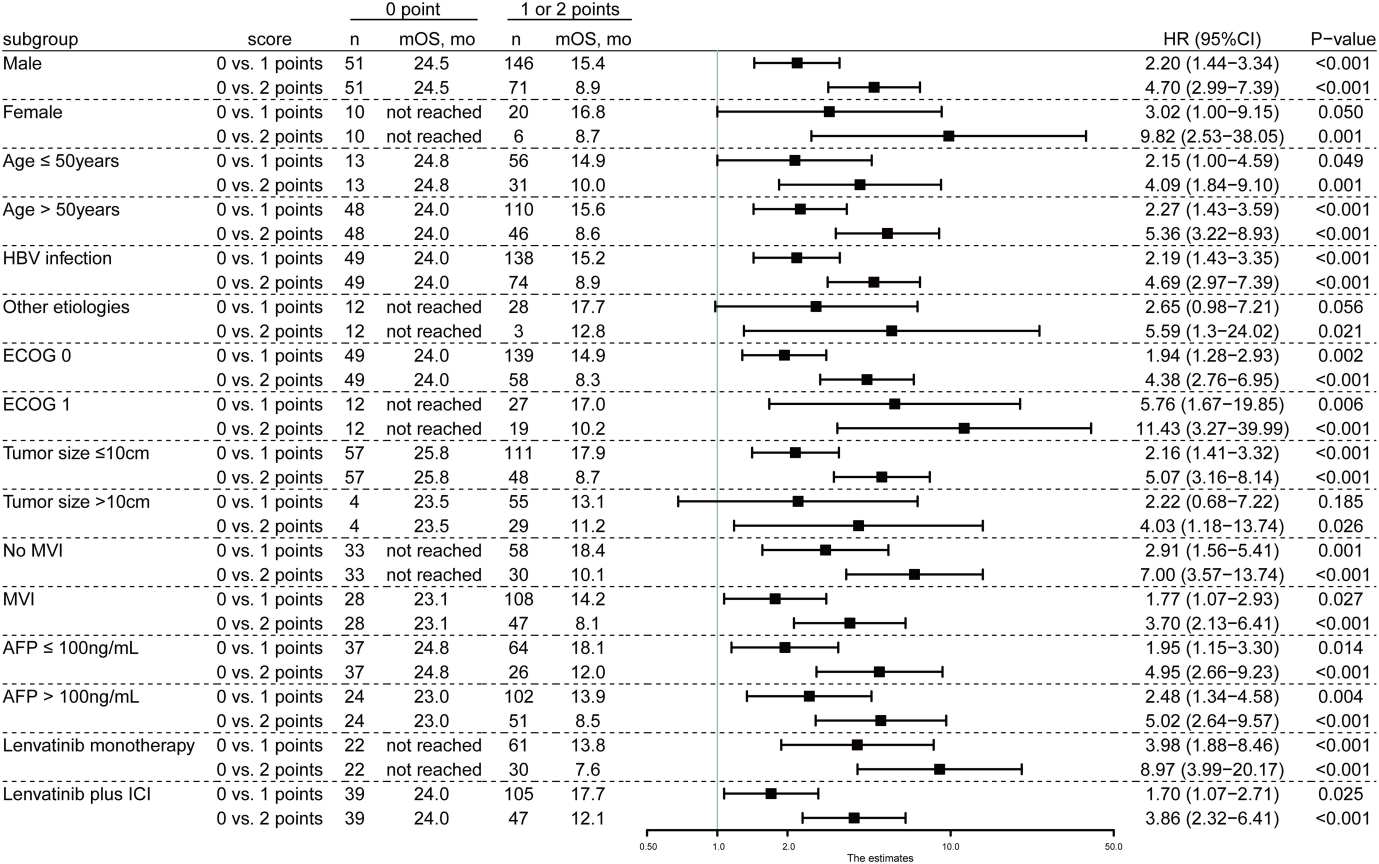
The prognostic efficiency of PIMET score in subgroups of patients receiving lenvatinib monotherapy or lenvatinib plus ICI for unresectable HCC. Median OS (Kaplan-Meier method) and hazard ratios (univariable Cox regression) for death comparing PIMET categories (0 vs. 1 and 0 vs. 2 points) in different subgroups in the whole cohort. AFP, alpha-fetoprotein; CI, confidence interval; ECOG, Eastern Cooperative Oncology Group performance status; HBV, hepatitis B virus; HCC, hepatocellular carcinoma; HR, hazard ratio; ICI, immune checkpoint inhibitor; mOS, median overall survival; MVI, macrovascular invasion.

To identify the predictive value of the model in different subgroup populations, subgroup analyses were applied to the whole cohort. As demonstrated in **Figure 3**, the PIMET score held predictive value regardless the gender, age, etiology, Eastern Cooperative Oncology Group (ECOG) performance status, tumor size, the presence of MVI, and alpha-fetoprotein (AFP) level.

Importantly, we investigated the prognostic efficiency of the PIMET score in patients receiving lenvatinib monotherapy and patients receiving combination therapy with ICI, respectively. In patients with lenvatinib monotherapy, median OS was not reached for PIMET-low (n=22), 13.8 months for PIMET-int (n=61, P<0.001), and 7.6 months for PIMET-high (n=30, P<0.001). In patients with lenvatinib plus ICI, the median OS was 24.0 months for PIMET-low (n=39), 17.7 months for PIMET-int (n=105, P=0.025), and 12.1 months for PIMET-high (n=47, P<0.001).

### PIMET score distinguishing patients with different treatment responses

The treatment responses in patients with different PIMET scores were demonstrated in **Table 3**. The ORR was 49.2% in the PIMET-low group, 30.1% in PIMET-int group, and 28.6% in PIMET-high group. The patients with PIMET-low showed significantly higher ORR compared with those with PIMET-int (P=0.012) and PIMET-high (P=0.021). And the DCR in the three groups was 80.3%, 82.5%, and 62.3%, respectively. Both the patients with PIMET-low (P=0.035) and the patients with PIMET-int (P=0.001) had higher DCR than those with PIMET-high.

**Table 3:** Treatment responses in HCC patients with different PIMET scores.

Response	PIMET-low (n=61)	PIMET-int (n=166)	PIMET-high (n=77)	*P*
**CR**	8 (13.1)	4 (2.4)	2 (2.6)	0.002
**PR**	22 (36.1)	46 (27.7)	20 (26.0)	0.376
**SD**	19 (31.1)	87 (52.4)	26 (33.8)	0.002
**PD**	12 (19.7)	29 (17.5)	29 (37.7)	0.002
**ORR**	30 (49.2)	50 (30.1)	22 (28.6)	0.015
**DCR**	49 (80.3)	137 (82.5)	48 (62.3)	0.002

CR, complete response; DCR, disease control rate; HCC, hepatocellular carcinoma; ORR, objective response rate; PD, progression disease; PR, partial response; SD, stable disease.

### PIMET score identifying patients who benefited from the lenvatinib plus ICI

**Figure 4:**
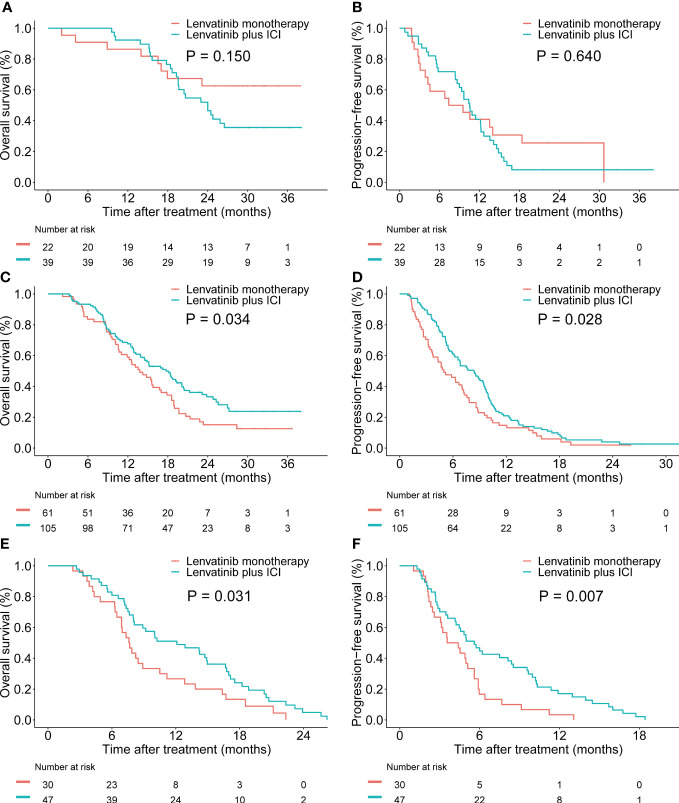
Efficacy of lenvatinib monotherapy and lenvatinib plus ICI in patients with unresectable HCC stratified by PIMET score. **(A)** The Kaplan-Meier survival curves of OS according to the treatment regimens in the PIMET-low group. **(B)** The Kaplan-Meier survival curves of PFS according to the treatment regimens in the PIMET-low group. **(C)** The Kaplan-Meier survival curves of OS according to the treatment regimens in the PIMET-int group. **(D)** The Kaplan-Meier survival curves of PFS according to the treatment regimens in the PIMET-int group. **(E)** The Kaplan-Meier survival curves of OS according to the treatment regimens in the PIMET-high group. **(F)** The Kaplan-Meier survival curves of PFS according to the treatment regimens in the PIMET-high group. HCC, hepatocellular carcinoma; ICI, immune checkpoint inhibitor; OS, overall survival; PFS, progression-free survival.

Furthermore, we investigated the association between the PIMET score and the prognosis of different treatment regimens. In the PIMET-low group, both the OS (P=0.150, **Figures 4A**) and PFS (P=0.640, **Figures 4B**) were similar between patients with lenvatinib monotherapy and patients with combination therapy. However, we found that in the PIMET-int group, the combination of lenvatinib with ICI significantly prolonged the OS (median OS: 17.7 vs 13.8 months, P=0.034, **Figure 4C**) and PFS (median PFS: 8.4 vs 5.0 months, P=0.028, **Figure 4D**) compared with lenvatinib monotherapy. Meanwhile, the lenvatinib plus ICI also improved outcomes of PIMET-high patients (median OS: 12.1 vs 7.6 months, P=0.031, **Figure 4E**; median PFS: 5.6 vs 4.0 months, P =0.007, **Figure 4F**).

## Discussion

In the present study, we constructed a prognostic model called PIMET score using PIVKA-II and metastasis status to predict the prognosis of patients with uHCC receiving lenvatinib monotherapy or lenvatinib plus ICI. Patients were classified as PIMET-high (with metastasis and PIVKA-II>600 mAU/mL), PIMET-int (with metastasis or PIVKA-II>600 mAU/mL) and PIMET-low (without metastasis and PIVKA-II <600 mAU/mL) accordingly. There were significantly distinct OS and PFS among patients in the three groups, both in the training cohort and validation cohort. The C-index of the PIMET score was 0.63 and 0.67 for survival prediction in the training cohort and validation cohort, respectively, indicating that the PIMET score could serve as a favorable prognostic biomarker for uHCC treated with lenvatinib monotherapy or lenvatinib plus ICI.

There were some established prognostic models for predicting the survival of patients who received systemic treatments for advanced HCCs, including sorafenib, lenvatinib, and immunotherapy (13, 17–20). The PROSASH and PROSASH-II model, which consisted of serum albumin, bilirubin, AFP, macrovascular invasion, extrahepatic spread, and largest tumor size, were built for survival prediction of patients with HCC treated with sorafenib. The C-index of the PROSASH and PROSASH-II models were 0.62 and 0.63 in clinical practice, respectively (17, 18). The CRAFITY score was constructed in patients with HCC undergoing immunotherapy. It comprised CRP and AFP and showed a C-index value of 0.62 in both the training and validation cohorts. However, the predicting model for both lenvatinib monotherapy and lenvatinib plus ICI was lacking. In the present study, we initially developed a general prognostic model for patients with uHCC treated with lenvatinib monotherapy or lenvatinib plus ICI, which achieved satisfactory performance. Importantly, subgroup analysis found that the PIMET score had similar predictive value in patients treated with lenvatinib monotherapy and combination therapy, indicating that the model was suitable for these two kinds of treatments.

Notably, we found that the combination therapy conferred survival benefits for patients with PIMET-int and PIMET-high, instead of those with PIMET-low. Patients with PIMET-int and PIMET-high treated with lenvatinib plus ICI showed significantly better prognoses than those treated with lenvatinib monotherapy. However, the combination therapy didn’t confer survival benefits to patients with PIMET-low. Several studies reported that lenvatinib plus ICI could exert unique immunomodulatory effects and confer better clinical benefits than monotherapy (21, 22). However, the recent LEAP-002 failed to meet the primary endpoint, indicating that not all patients could benefit from combination therapy. Consistently, we also found that compared with lenvatinib monotherapy, lenvatinib plus ICI could improve outcomes of uHCC patients with risk factors, such as PIVKA-II > 600 mAU/mL or the presence of metastasis. For those with PIVKA-II ≤ 600 mAU/mL and without metastasis, the combination of lenvatinib and ICI showed similar survival benefits with lenvatinib monotherapy. One reasonable explanation was that patients with PIMET-low showed favorable outcomes after receiving the lenvatinib monotherapy and thus the efficacy of combined immunotherapy was limited. To our knowledge, the PIMET score is the first prognostic model to guide the clinical treatment decision and facilitate precision medicine for uHCC patients.

In the present study, we identified that PIVKA-II could predict the survival of uHCC patients receiving lenvatinib monotherapy or lenvatinib plus ICI. The prognostic significance of PIVKA-II was controversial according to previous studies. Many literatures showed that the PIVKA-II could predict prognosis and serve as a biomarker for patients who received locoregional therapy (23–25). However, some studies reported that PIVKA-II could not distinguish patients with a high risk of recurrence after curative resection (26, 27). What’s more, there was no report on the application of PIVKA-II in systemic treatment. In the present study, we for the first time, found that PIVKA-II could serve as a strong indicator of prognosis for uHCC.

There were several limitations in the present study. The most prominent is the retrospective design since this is subject to unintentional biases. Thus, a prospective study was needed to validate the prognostic model. Besides, although all included patients were treated with lenvatinib, some patients also applied ICI simultaneously, potentially leading to a selection bias. To account for a potential selection bias, we performed subgroup analyses and successfully validated the PIMET score in different treatment strategies. In addition, the population characteristics of lenvatinib monotherapy and ICI combination therapy might not be equal and thus to further validate the efficacy of lenvatinib plus ICI, a randomized control clinical trial in patients with PIMET-int or PIMET-high was needed. Finally, the most common etiology in our study was the hepatitis B virus. Whether the PIMET score could achieve a similar efficacy for patients with other etiology such as hepatitis C virus needs further study.

In conclusion, we built the PIMET score, which comprised PIVKA-II and metastasis status, to predict the prognosis of patients with uHCC. The PIMET score showed prognostic value in patients receiving lenvatinib monotherapy and lenvatinib plus ICI. Patients with PIMET-int or PIMET-high could benefit from the combination of lenvatinib and ICI. The above results indicated that this model could be widely used in clinical practice and facilitated the decision-making of treatment strategies for uHCC patients.

## Data availability statement

The raw data supporting the conclusions of this article will be made available by the authors, without undue reservation.

## Ethics statement

The studies involving human participants were reviewed and approved by the ethics committee of Zhongshan hospital. Written informed consent for participation was not required for this study in accordance with the national legislation and the institutional requirements.

## Author contributions

D-ZG, S-YZ, AH and JZ designed the study and wrote the manuscript. D-ZG, S-YD and AH performed analysis of the data. X-RY, J-YY and Y-PW collected patients’ information and created database. JZ, YC, JF and S-XR interpreted the data and revised the manuscript. All authors edited the manuscript. All authors contributed to the article and approved the submitted version.

## References

[1] EASL Clinical Practice Guidelines. Management of hepatocellular carcinoma. J Hepatol (2018) 69(1):182–236. doi: 10.1016/j.jhep.2018.03.019 29628281

[2] Siegel RL Miller KD Fuchs HE Jemal A. Cancer statistics, 2022. CA: Cancer J Clin (2022) 72(1):7–33. doi: 10.3322/caac.21708 35020204

[3] Llovet JM Kelley RK Villanueva A Singal AG Pikarsky E Roayaie S. Hepatocellular carcinoma. Nat Rev Dis Primers (2021) 7(1):6. doi: 10.1038/s41572-020-00240-3 33479224 PMC13537433

[4] Cabibbo G Enea M Attanasio M Bruix J Craxì A Cammà C. A meta-analysis of survival rates of untreated patients in randomized clinical trials of hepatocellular carcinoma. Hepatol (Baltimore Md) (2010) 51(4):1274–83. doi: 10.1002/hep.23485 20112254

[5] Kudo M Finn RS Qin S Han KH Ikeda K Piscaglia F. Lenvatinib versus sorafenib in first-line treatment of patients with unresectable hepatocellular carcinoma: A randomised phase 3 non-inferiority trial. Lancet (London England) (2018) 391(10126):1163–73. doi: 10.1016/s0140-6736(18)30207-1 29433850

[6] Arance A de la Cruz-Merino L Petrella TM Jamal R Ny L Carneiro A. Phase II LEAP-004 study of lenvatinib plus pembrolizumab for melanoma with confirmed progression on a programmed cell death protein-1 or programmed death ligand 1 inhibitor given as monotherapy or in combination. J Clin Oncol Off J Am Soc Clin Oncol (2022) 41(1):75–85. doi: 10.1200/jco.22.00221 35867951

[7] Motzer R Porta C Alekseev B Rha SY Choueiri TK Mendez-Vidal MJ. Health-related quality-of-life outcomes in patients with advanced renal cell carcinoma treated with lenvatinib plus pembrolizumab or everolimus versus sunitinib (CLEAR): A randomised, phase 3 study. Lancet Oncol (2022) 23(6):768–80. doi: 10.1016/s1470-2045(22)00212-1 PMC10284118 35489363

[8] Makker V Colombo N Casado Herráez A Santin AD Colomba E Miller DS. Lenvatinib plus pembrolizumab for advanced endometrial cancer. New Engl J Med (2022) 386(5):437–48. doi: 10.1056/NEJMoa2108330 PMC11651366 35045221

[9] Motzer R Alekseev B Rha SY Porta C Eto M Powles T. Lenvatinib plus pembrolizumab or everolimus for advanced renal cell carcinoma. New Engl J Med (2021) 384(14):1289–300. doi: 10.1056/NEJMoa2035716 33616314

[10] Hiraoka A Kumada T Tada T Fukunishi S Atsukawa M Hirooka M. Nutritional index as prognostic indicator in patients receiving lenvatinib treatment for unresectable hepatocellular carcinoma. Oncology (2020) 98(5):295–302. doi: 10.1159/000506293 32097925

[11] Rimini M Kang W Burgio V Persano M Aoki T Shimose S. Validation of the easy-to-use lenvatinib prognostic index to predict prognosis in advanced hepatocellular carcinoma patients treated with lenvatinib. Hepatol Res Off J Japan Soc Hepatol (2022) 52(12):1050–9. doi: 10.1111/hepr.13824 35960789

[12] Kariyama K Hiraoka A Kumada T Yasuda S Toyoda H Tsuji K. Chronological change in serum albumin as a prognostic factor in patients with hepatocellular carcinoma treated with lenvatinib: Proposal of albumin simplified grading based on the modified albumin-bilirubin score (ALBS grade). J Gastroenterol (2022) 57(8):581–6. doi: 10.1007/s00535-022-01883-7 35763116

[13] Scheiner B Pomej K Kirstein MM Hucke F Finkelmeier F Waidmann O. Prognosis of patients with hepatocellular carcinoma treated with immunotherapy - development and validation of the CRAFITY score. J Hepatol (2022) 76(2):353–63. doi: 10.1016/j.jhep.2021.09.035 34648895

[14] Reig M Forner A Rimola J Ferrer-Fàbrega J Burrel M Garcia-Criado Á. BCLC strategy for prognosis prediction and treatment recommendation: The 2022 update. J Hepatol (2022) 76(3):681–93. doi: 10.1016/j.jhep.2021.11.018 PMC8866082 34801630

[15] Zhou J Sun H Wang Z Cong W Wang J Zeng M. Guidelines for the diagnosis and treatment of hepatocellular carcinoma (2019 edition). Liver Cancer (2020) 9(6):682–720. doi: 10.1159/000509424 33442540 PMC7768108

[16] Lencioni R Llovet JM. Modified RECIST (mRECIST) assessment for hepatocellular carcinoma. Semin Liver Dis (2010) 30(1):52–60. doi: 10.1055/s-0030-1247132 20175033 PMC12268942

[17] Berhane S Fox R García-Fiñana M Cucchetti A Johnson P. Using prognostic and predictive clinical features to make personalised survival prediction in advanced hepatocellular carcinoma patients undergoing sorafenib treatment. Br J Cancer (2019) 121(2):117–24. doi: 10.1038/s41416-019-0488-4 PMC6738086 31182766

[18] Labeur TA Berhane S Edeline J Blanc JF Bettinger D Meyer T. Improved survival prediction and comparison of prognostic models for patients with hepatocellular carcinoma treated with sorafenib. Liver Int Off J Int Assoc Study Liver (2020) 40(1):215–28. doi: 10.1111/liv.14270 PMC6973249 31579990

[19] Sansone V Tovoli F Casadei-Gardini A Di Costanzo GG Magini G Sacco R. Comparison of prognostic scores in patients with hepatocellular carcinoma treated with sorafenib. Clin Trans Gastroenterol (2021) 12(1):e00286. doi: 10.14309/ctg.0000000000000286 PMC7808555 33443944

[20] Demirtas CO Ricco G Ozdogan OC Baltacioglu F Ones T Yumuk PF. Proposal and validation of a novel scoring system for hepatocellular carcinomas beyond curability borders. Hepatol Commun (2022) 6(3):633–45. doi: 10.1002/hep4.1836 PMC8870011 34751001

[21] Torrens L Montironi C Puigvehí M Mesropian A Leslie J Haber PK. Immunomodulatory effects of lenvatinib plus anti-programmed cell death protein 1 in mice and rationale for patient enrichment in hepatocellular carcinoma. Hepatol (Baltimore Md) (2021) 74(5):2652–69. doi: 10.1002/hep.32023 PMC12155127 34157147

[22] Yi C Chen L Lin Z Liu L Shao W Zhang R. Lenvatinib targets FGF receptor 4 to enhance antitumor immune response of anti-programmed cell death-1 in HCC. Hepatol (Baltimore Md) (2021) 74(5):2544–60. doi: 10.1002/hep.31921 34036623

[23] Nagaoka S Yatsuhashi H Hamada H Yano K Matsumoto T Daikoku M. The des-gamma-carboxy prothrombin index is a new prognostic indicator for hepatocellular carcinoma. Cancer (2003) 98(12):2671–7. doi: 10.1002/cncr.11839 14669288

[24] Kobayashi M Ikeda K Kawamura Y Yatsuji H Hosaka T Sezaki H. High serum des-gamma-carboxy prothrombin level predicts poor prognosis after radiofrequency ablation of hepatocellular carcinoma. Cancer (2009) 115(3):571–80. doi: 10.1002/cncr.24031 19117347

[25] Toyoda H Kumada T Kaneoka Y Osaki Y Kimura T Arimoto A. Prognostic value of pretreatment levels of tumor markers for hepatocellular carcinoma on survival after curative treatment of patients with HCC. J Hepatol (2008) 49(2):223–32. doi: 10.1016/j.jhep.2008.04.013 18571271

[26] Toyoda H Kumada T Tada T Niinomi T Ito T Kaneoka Y. Prognostic significance of a combination of pre- and post-treatment tumor markers for hepatocellular carcinoma curatively treated with hepatectomy. J Hepatol (2012) 57(6):1251–7. doi: 10.1016/j.jhep.2012.07.018 22824818

[27] Tateishi R Shiina S Yoshida H Teratani T Obi S Yamashiki N. Prediction of recurrence of hepatocellular carcinoma after curative ablation using three tumor markers. Hepatol (Baltimore Md) (2006) 44(6):1518–27. doi: 10.1002/hep.21408 17133456

