# Supplementary material for: Prognostic model for predicting outcome and guiding treatment decision for unresectable hepatocellular carcinoma treated with lenvatinib monotherapy or lenvatinib plus immunotherapy
Source: Front Immunol. 2023 Feb 23;14:1141199. doi: 10.3389/fimmu.2023.1141199 (PMC9995378; doi:10.3389/fimmu.2023.1141199)

Supplementary Figure 1. The predicting value of PIMET score for patients receiving lenvatinib monotherapy or lenvatinib plus ICI for unresectable HCC.

(A) The Kaplan-Meier survival curves of PFS according to PIMET score in the training cohort. (B) The AUCs of PIMET score for predicting 6-, 12-, and 24-month PFS in the training cohort. (C) The Kaplan-Meier survival curves of PFS according to PIMET score in the validation cohort. (D) The AUCs of PIMET score for predicting 6-, 12-, and 24-month PFS in the validation cohort.

AUC, area under the receiver-operating characteristic curve; HCC, hepatocellular carcinoma; PFS, progression-free survival.

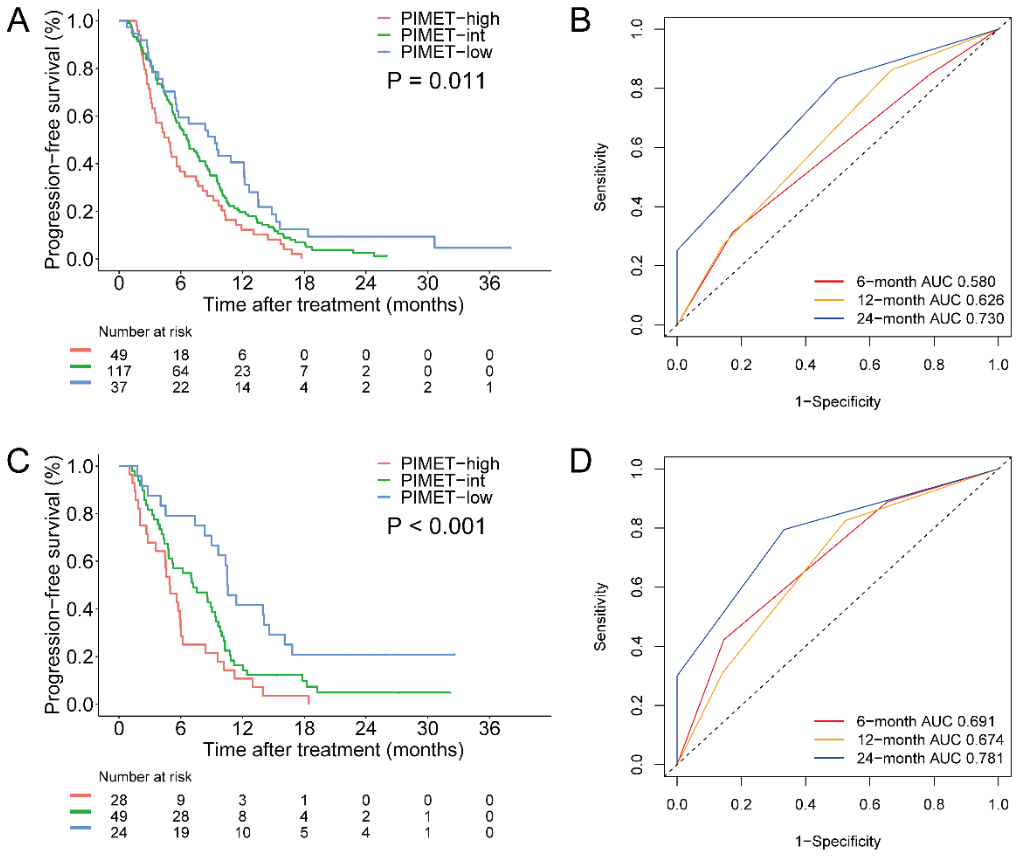

Supplement: Supplementary file 1 [file DataSheet_1.pdf]
